# Comparison of YouTube Videos and Artificial Intelligence Chatbot Responses Regarding Pediatric Pelvic Floor Dysfunction: A Cross-Sectional Infodemiology Study

**DOI:** 10.7759/cureus.112387

**Published:** 2026-07-10

**Authors:** Volkan Altinok, Ecem I Altinok

**Affiliations:** 1 Department of Pediatric Surgery, Ordu Üniversitesi, Ordu, TUR; 2 Department of Pediatrics, Ordu Üniversitesi, Ordu, TUR

**Keywords:** ai-based chatbots, artificial intelligence in medicine, dysfunctional voiding, pediatrics and child health, pelvic floor dysfunction, pelvic floor exercise, youtube videos

## Abstract

Background: Pediatric pelvic floor dysfunction is a common cause of lower urinary tract symptoms and bowel dysfunction in children, requiring accurate and reliable educational resources for patients and caregivers. With the increasing use of online health information, both YouTube (Google LLC, Mountain View, CA, USA) and artificial intelligence (AI) chatbots have become frequently used sources of medical guidance. This study aimed to evaluate and compare the educational quality, reliability, and safety of YouTube videos and AI-generated responses related to pediatric pelvic floor dysfunction.

Materials and methods: In this cross-sectional infodemiology study, YouTube was searched using five predefined pediatric pelvic floor-related search terms, and the first 50 videos for each term were screened. After duplicate removal and eligibility assessment, 25 videos were included for analysis. Three publicly available AI chatbots (ChatGPT (OpenAI, San Francisco, CA, USA), Gemini (Google LLC, Mountain View, CA, USA), and Copilot (Microsoft Corporation, Redmond, WA, USA)) were evaluated using a standardized set of 20 pediatric pelvic floor-related questions. Educational quality was independently assessed using the DISCERN instrument, the Global Quality Scale (GQS), and the Pediatric Pelvic Floor Education and Reliability Score (PPFERS), a pediatric-specific assessment tool developed for this study.

Results: The 25 included YouTube videos accumulated 447,398 total views. Physiotherapists or pediatric physical therapists were the most common content creators (44.0%), followed by healthcare institutions (28.0%). The mean DISCERN, GQS, and PPFERS scores for YouTube videos were 66.24 ± 8.64, 4.28 ± 0.74, and 16.88 ± 1.96, respectively. Among AI chatbots, Gemini achieved the highest educational quality scores (76.00 ± 1.92, 4.95 ± 0.22, and 19.35 ± 0.59), followed by ChatGPT (74.20 ± 2.40, 4.85 ± 0.37, and 18.60 ± 0.82) and Copilot (67.85 ± 2.03, 4.35 ± 0.49, and 17.10 ± 0.72). Overall, AI-generated responses demonstrated higher mean educational quality scores than the YouTube video cohort.

Conclusions: Both YouTube videos and AI-generated responses provided generally useful educational information regarding pediatric pelvic floor dysfunction. However, AI chatbots, particularly Gemini and ChatGPT, demonstrated greater educational consistency and closer alignment with contemporary pediatric urotherapy principles. AI-assisted educational tools may serve as valuable adjuncts to patient and caregiver education but should complement, rather than replace, professional medical evaluation and individualized clinical guidance.

## Introduction

Pediatric pelvic floor dysfunction encompasses a spectrum of lower urinary tract and bowel-related disorders, including dysfunctional voiding, daytime urinary incontinence, urgency-frequency symptoms, nocturnal enuresis, and bladder-bowel dysfunction. These conditions may negatively affect children's quality of life, school participation, psychosocial well-being, and family functioning. Beyond symptom burden, pediatric pelvic floor dysfunction may also lead to repeated healthcare visits, caregiver anxiety, treatment frustration, and reduced adherence to long-term conservative management. Conservative management strategies, including urotherapy, bladder training, bowel management, pelvic floor muscle education, and biofeedback-assisted rehabilitation, represent the cornerstone of first-line treatment for many children with functional lower urinary tract symptoms [1,2].

Successful conservative treatment requires accurate, age-appropriate, and comprehensible education for both children and caregivers. Unlike adult pelvic floor rehabilitation, pediatric pelvic floor therapy focuses not only on muscle strengthening but also on improving pelvic floor awareness, relaxation, coordination during voiding, toilet posture, bladder habits, bowel management, and adherence to daily behavioral recommendations. Therefore, the quality of educational information has direct clinical relevance. Incorrect instructions, excessive emphasis on muscle contraction, breath-holding during exercises, repeated interruption of urinary flow, or failure to explain relaxation and voiding coordination may reinforce dysfunctional voiding patterns, reduce treatment effectiveness, delay professional evaluation, and undermine adherence to clinician-supervised urotherapy [1,2].

With the increasing use of digital health platforms, parents and caregivers frequently seek medical information from online sources before or after clinical consultations. In real-world practice, these digital resources may shape parental expectations, home-based exercise practices, help-seeking behavior, and adherence to treatment recommendations. YouTube (Google LLC, Mountain View, CA, USA) has become one of the most commonly accessed sources of health-related information because it provides visual demonstrations and practical explanations that may be easier to understand than written materials [3,4]. However, the same features that make YouTube accessible may also increase the influence of inaccurate, incomplete, or non-guideline-consistent content. Previous studies evaluating YouTube videos related to pelvic floor muscle training and pelvic floor rehabilitation have demonstrated considerable variability in educational quality, reliability, and information completeness [5-8]. Similar concerns have been reported in studies of online urological content, in which highly viewed videos do not necessarily provide accurate or guideline-consistent information.

Simultaneously, artificial intelligence (AI)-based chatbots powered by large language models, including ChatGPT (OpenAI, San Francisco, CA, USA), Gemini (Google LLC, Mountain View, CA, USA), and Copilot (Microsoft Corporation, Redmond, WA, USA), are increasingly being used by the public to obtain medical information [9]. Recent studies conducted in urology and urogynecology have shown that AI systems can generate structured, comprehensive, and generally high-quality educational responses [9-12]. Nevertheless, their real-world use raises important safety concerns. AI-generated information may appear authoritative even when it is incomplete, insufficiently individualized, or inconsistent with evidence-based clinical guidance. Additionally, text-based AI responses may be limited in their ability to demonstrate the visual and interactive components central to pediatric urotherapy, such as toilet posture, breathing coordination, pelvic floor relaxation, and proper exercise performance. These concerns may be particularly important in pediatric pelvic floor dysfunction, where inappropriate advice could delay professional evaluation, overlook warning signs, encourage incorrect exercise techniques, or create unrealistic expectations about self-directed treatment without clinical supervision [13].

Despite the growing availability of YouTube-based educational content and AI-generated health information, the quality and reliability of information on pediatric pelvic floor dysfunction remain underexplored. This represents an important real-world gap because clinicians are increasingly required to counsel families not only about treatment but also about how to interpret and safely use digital health information encountered outside the clinical setting. Evaluating these resources may help identify gaps in educational content, clarify the relative strengths and limitations of different digital modalities, and guide clinicians in directing families toward safer, more reliable, and developmentally appropriate educational materials. Such evaluation may also help clinicians explain why online resources and AI chatbots should be used only as complementary educational supports and not as substitutes for individualized, clinician-supervised urotherapy.

A further methodological challenge is that YouTube videos and AI chatbot responses represent fundamentally different educational modalities. YouTube videos are unprompted, heterogeneous, video-based materials that may include visual demonstrations but vary widely in structure, source credibility, and completeness. In contrast, AI chatbot responses are text-based outputs generated under standardized prompting conditions and may appear more organized because they directly respond to curated questions. Therefore, any comparison between these modalities should be interpreted within the study's structured assessment framework rather than as evidence of real-world clinical superiority of one modality over the other. Moreover, AI-generated responses represent a temporal snapshot, as large language model backends may change over time through non-transparent updates.

We are unaware of any previous studies that directly compare YouTube videos and AI chatbot responses regarding pediatric pelvic floor dysfunction using a pediatric-specific evaluation framework. Therefore, this study aimed to compare two distinct educational modalities, video-based YouTube content and text-based AI chatbot responses generated under standardized prompting conditions, regarding pediatric pelvic floor dysfunction and pelvic floor exercises using DISCERN, the Global Quality Scale (GQS), and the study-specific Pediatric Pelvic Floor Education and Reliability Score (PPFERS). The primary research question was whether AI-generated responses and YouTube videos differ in educational quality, reliability, safety-related content, and completeness of pediatric urotherapy-oriented education. In this study, consistency with guideline-based pediatric urotherapy principles was assessed as a structured content domain within the PPFERS checklist. It was not intended to represent a direct item-by-item International Children’s Continence Society (ICCS) guideline-adherence analysis.

## Materials and methods

Study design

This cross-sectional infodemiology study was conducted to evaluate and compare the quality, reliability, safety, and educational value of online information related to pediatric pelvic floor dysfunction and its conservative management. The study consisted of two complementary components: an analysis of YouTube video content and an evaluation of text-based responses generated by AI chatbots under standardized prompting conditions. Because the study exclusively analyzed publicly available online content and did not involve human participants, patient data, or identifiable personal information, institutional ethics committee approval was not required.

YouTube searches were performed on May 24, 2026, using Google Chrome (Google LLC, Mountain View, CA, USA) in incognito mode to minimize personalization bias and reduce the influence of browsing history, user preferences, and algorithm-based recommendations. No user account was logged into YouTube during the search process. Five search terms were selected based on commonly used terminology related to pediatric pelvic floor dysfunction and conservative management: “pediatric pelvic floor exercises,” “pelvic floor exercises for children,” “pediatric pelvic floor therapy,” “dysfunctional voiding exercises child,” and “bladder training child.” Videos were sorted according to relevance using YouTube’s default search algorithm.

Because this was a cross-sectional infodemiology study based on publicly available online content, a formal power-based sample size calculation was not applicable. Therefore, the sample size was determined according to the predefined search strategy. The first 50 videos identified for each search term were screened, resulting in a total of 250 videos for initial assessment. The first 50 videos were selected to obtain a broad sample of videos ranked highly by YouTube’s relevance-based search algorithm while maintaining a screening strategy that reflects content likely to be encountered by users during routine online searches. This approach was intended to approximate typical user exposure to easily accessible YouTube content; however, it may not fully represent all possible user behaviors, as some users may view fewer videos, use different search terms, apply different sorting filters, or be influenced by personalized recommendations.

Duplicate videos identified across multiple search terms were removed before eligibility assessment. Video titles, thumbnails, and descriptions were initially screened for relevance, after which potentially eligible videos underwent full-content review. Videos were included if they were presented in English, included audio narration, provided educational information on pediatric pelvic floor dysfunction or its conservative management, and were publicly accessible at the time of evaluation. Videos were excluded if they primarily consisted of advertisements or promotional content, focused exclusively on adult pelvic floor disorders, contained unrelated information, lacked audio narration, were shorter than 30 seconds, or represented duplicate uploads.

For each included video, duration, number of views, likes, comments, and time since upload were recorded. Uploader characteristics were categorized as physiotherapists/pediatric physical therapists, healthcare institutions, physicians/urologists, or independent health-information channels.

AI selection, platform settings, and question development

Three publicly accessible free large language model-based AI chatbots were evaluated in this study: ChatGPT (free public web interface), Gemini (Gemini Flash mode, free public web interface), and Copilot (Smart mode, free public web interface). All chatbot evaluations were conducted using the publicly available free versions of the respective platforms on May 24, 2026. No paid subscription, user-specific customization, memory function, or prior conversation history was used during the evaluation. Each chatbot was accessed in a new session, and the same standardized questions were entered independently into each platform.

For ChatGPT, the free public web interface was used; however, the exact backend model identifier was not consistently displayed or recorded during evaluation and was therefore reported as “not specified by the platform interface.” For Gemini, the Flash mode displayed in the interface was used. For Copilot, the Smart mode displayed in the interface was used. No additional web browsing or internet search functions were manually activated beyond the default settings of the free public interfaces. When web-connected response generation was part of the platform’s default public mode, this default setting was left unchanged and recorded as such. The chatbot outputs were recorded as a cross-sectional snapshot of platform performance on the evaluation date. Because large language model backends may be updated over time and exact backend identifiers are not always transparently displayed in free public interfaces, the responses were interpreted as time-specific outputs rather than fixed or permanent platform characteristics.

A standardized set of 20 questions was developed primarily according to contemporary pediatric urotherapy principles, relevant clinical guidance, and expert clinical judgment. The questions addressed pediatric pelvic floor exercises, dysfunctional voiding, bladder training, urotherapy, daytime urinary incontinence, nocturnal enuresis, constipation-related urinary symptoms, exercise safety, pelvic floor muscle identification, and parent-directed educational guidance. Common educational themes observed during the YouTube content review were considered only to ensure that the question set reflected topics likely to be encountered by caregivers and patients during routine online searches; they were not used as the primary basis for constructing the chatbot assessment framework.

Educational quality assessment

Educational quality was evaluated using three complementary assessment tools. The DISCERN instrument, originally developed by Charnock et al. to assess the quality and reliability of consumer health information on treatment choices, was used to evaluate the reliability and quality of educational content [14]. DISCERN consists of 16 items scored on a five-point Likert scale, producing total scores ranging from 16 to 80, with higher scores indicating greater reliability and educational quality.

Overall educational quality was additionally assessed using the 5-point GQS, a previously published instrument widely used in studies evaluating online medical information [15].

The authors developed the PPFERS as a study-specific, pediatric-focused structured scoring checklist to assess whether YouTube videos and AI-generated responses included key elements of evidence-based education on pediatric pelvic floor dysfunction and pelvic floor exercises. The PPFERS was not intended to serve as a formally validated psychometric instrument, but rather as an exploratory, content-based checklist for comparative assessment within the scope of this study.

Items were generated after reviewing recommendations from the ICCS, contemporary pediatric urotherapy guidance, and previously published online health-information assessment studies [1,2]. The draft checklist was reviewed by clinicians experienced in pediatric healthcare, pediatric urinary symptoms, and pediatric surgical/urological conditions. Items were revised for clinical relevance, clarity, and applicability to both video-based and AI-generated educational content. A pilot assessment was then conducted on sample materials not included in the final analysis, and minor wording modifications were made before final scoring.

The final PPFERS included ten educational domains: scientific accuracy, child-friendly explanation, correct exercise instruction, avoidance of potentially harmful recommendations, breathing guidance, exercise frequency recommendations, relevance to pediatric urinary symptoms, recommendations for medical evaluation when appropriate, consistency with guideline-based pediatric urotherapy principles, and overall educational usefulness. Each domain was scored from 0 to 2, yielding a total score from 0 to 20, with higher scores indicating more complete, clinically appropriate, and safer pediatric pelvic floor education. The complete scoring criteria are provided in the Appendices.

Because the PPFERS items represent different formative content domains rather than interchangeable reflective items, internal consistency was not considered the primary reliability metric; instead, inter-rater reliability for the total PPFERS score was assessed using an intraclass correlation coefficient (ICC), as described below. The PPFERS has not undergone formal external validation, and this is acknowledged as a methodological limitation. As the instrument was newly developed by the authors for this study, no copyright permission was required.

Reviewer assessment

All included YouTube videos and AI-generated responses were independently evaluated by two reviewers with relevant clinical experience. The first reviewer was a pediatrician with experience in pediatric healthcare and pediatric urinary symptoms, and the second reviewer was a pediatric surgeon with experience in pediatric surgical and urotherapy-related conditions.

Before formal scoring, both reviewers reviewed the scoring instructions for DISCERN, GQS, and PPFERS. A calibration session was conducted using sample materials not included in the final analysis to ensure a shared understanding of the scoring criteria and minimize subjective variation. After calibration, all eligible materials were scored independently by both reviewers. Disagreements were subsequently resolved through discussion and consensus, and consensus scores were used for the final comparative analyses.

Inter-rater reliability was assessed using the reviewers’ initial independent scores before consensus resolution. For DISCERN and PPFERS total scores, inter-rater reliability was evaluated using two-way random-effects, absolute-agreement, single-measure ICCs. For the ordinal 5-point GQS score, inter-rater agreement was assessed using quadratic weighted Cohen’s kappa.

Statistical analysis

Statistical analyses were performed using SPSS Statistics version 26.0 (IBM Corp. Released 2019. IBM SPSS Statistics for Windows, Version 26.0. Armonk, NY: IBM Corp.). Continuous variables were assessed for normality using the Shapiro-Wilk test and visual inspection of histograms. Normally distributed variables are presented as mean ± standard deviation (SD), whereas non-normally distributed variables are presented as median and interquartile range (IQR). Categorical variables are presented as numbers (n) and percentages.

Inter-rater reliability for DISCERN and PPFERS total scores was evaluated using two-way random-effects absolute-agreement single-measures ICCs with 95% CIs. ICC values were interpreted as follows: values below 0.50 indicated poor reliability; 0.50-0.75, moderate reliability; 0.75-0.90, good reliability; and values above 0.90, excellent reliability. For the ordinal 5-point GQS score, inter-rater agreement was evaluated using quadratic weighted Cohen’s kappa.

Comparisons of educational quality scores, including DISCERN, GQS, and PPFERS, between the YouTube video cohort and the three AI chatbot groups (ChatGPT, Gemini, and Copilot) were performed using the Kruskal-Wallis test because the data were non-normally distributed and the group sample sizes were unequal. For significant Kruskal-Wallis test results, pairwise post-hoc comparisons were subsequently performed using Dunn’s test with Bonferroni correction to adjust for multiple comparisons. A two-sided p-value < 0.05 was considered statistically significant.

## Results

Video selection

**Figure 1:**
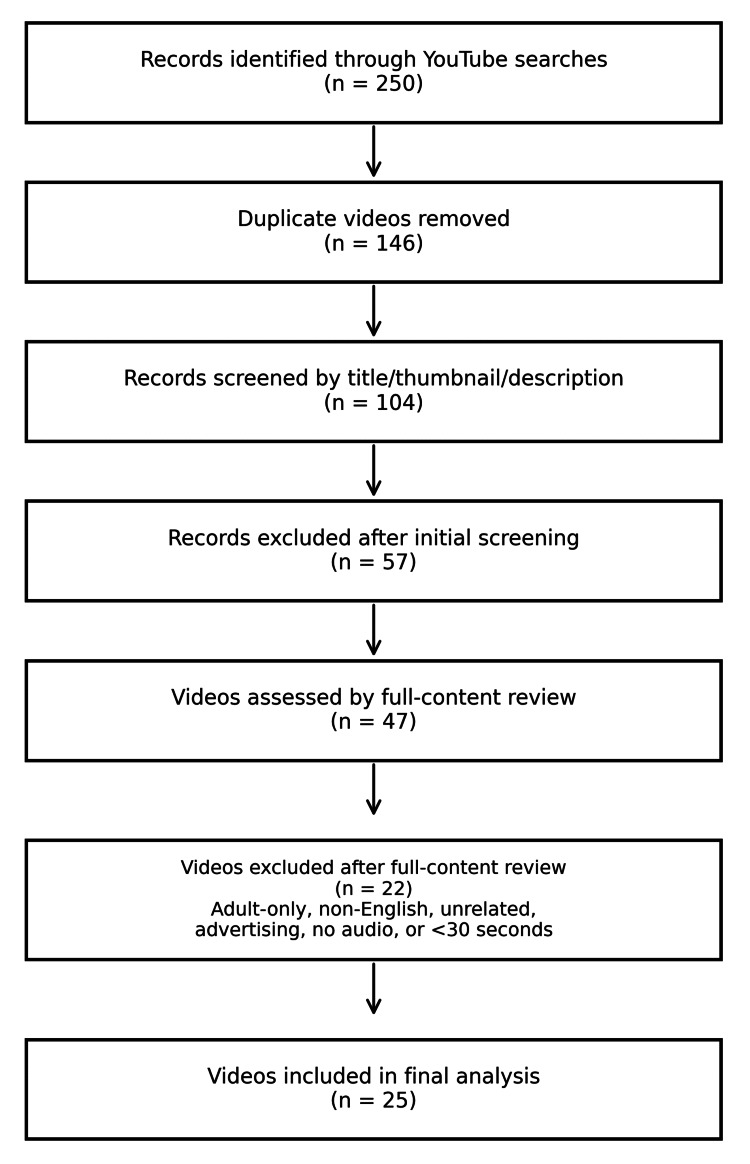
Flow diagram of YouTube video selection A total of 250 videos identified through five predefined YouTube search terms were screened for eligibility. After removal of duplicate videos, title, thumbnail, and description screening, and full content review, 25 videos met the predefined inclusion criteria and were included in the final analysis. Videos were excluded because they were duplicates, focused exclusively on adult populations, were not presented in English, contained unrelated or promotional content, lacked audio narration, or were shorter than 30 seconds.

A total of 250 videos were identified through five predefined search terms. After duplicate removal, title and description screening, and full-content eligibility assessment, 25 videos met the predefined inclusion criteria and were included in the final analysis. The detailed video selection process is illustrated in Figure 1.

Characteristics of included videos

The 25 included videos accumulated a total of 447,398 views, 1,178 likes, and 46 comments. Video duration ranged from 1.0 to 82.0 minutes, with a median duration of 3.8 minutes (IQR: 2.5-6.4). The median number of views was 1,100 (IQR: 419-3,300), although substantial variability was observed among videos, with view counts ranging from 123 to 198,036. Detailed characteristics of the included videos are summarized in Table 1.

**Table 1:** Characteristics of included YouTube videos Data are presented as numbers (n) or medians (IQR). IQR: interquartile range

Variable	Value
Number of included videos, n	25
Median views (IQR)	1,100 (419-3,300)
Range of views	123-198,036
Median duration, min (IQR)	3.8 (2.5-6.4)
Range of duration, min	1.0-82.0

Physiotherapists and pediatric physical therapists were the largest group of uploader types, accounting for 11/25 (44.0%) of included videos. Healthcare institutions contributed 7/25 (28.0%) videos, followed by independent health-information channels (5/25, 20.0%) and physicians/urologists (2/25, 8.0%) (Table 2).

**Table 2:** Distribution of uploaders Data are presented as numbers (n) and percentages (%).

Uploader type	n (%)
Physiotherapist/pediatric physical therapist	11 (44.0)
Healthcare institution	7 (28.0)
Independent health-information channel	5 (20.0)
Physician/urologist	2 (8.0)

Inter-rater reliability

Inter-rater reliability for the DISCERN total score was excellent, with a two-way random-effects, absolute-agreement, single-measures ICC of 0.98. Agreement for the ordinal GQS score was moderate, with a quadratic weighted Cohen’s kappa of 0.45. Inter-rater reliability for the total PPFERS score was good, with a two-way random-effects, absolute-agreement, single-measure ICC of 0.84 (95% CI 0.72-0.91).

Educational quality of YouTube videos

Overall educational quality of the included YouTube videos was moderate to high. The YouTube cohort achieved mean DISCERN, GQS, and PPFERS scores of 66.24 ± 8.64, 4.28 ± 0.74, and 16.88 ± 1.96, respectively. According to DISCERN classification criteria, 12/25 (48.0%) videos were classified as excellent quality, 10/25 (40.0%) as good quality, and 3/25 (12.0%) as fair quality. No videos were classified as poor or very poor. According to the PPFERS classification system, 9/25 (36.0%) videos were categorized as high quality, 12/25 (48.0%) as moderate quality, and 4/25 (16.0%) as low quality.

Educational quality of AI chatbot responses and comparison with YouTube videos

All three evaluated AI chatbots successfully generated responses to the predefined 20-question assessment set. Comparative educational quality scores for the YouTube video cohort and the evaluated AI chatbots are summarized in Table 3.

**Table 3:** Comparison of educational quality scores between YouTube videos and AI chatbots Data are presented as mean ± SD. Group comparisons were performed using the Kruskal-Wallis test. Statistical significance was defined as p < 0.05. SD: standard deviation, AI: artificial intelligence

Group	n	DISCERN (mean ± SD)	GQS (mean ± SD)	PPFERS (mean ± SD)
YouTube	25	66.24 ± 8.64	4.28 ± 0.74	16.88 ± 1.96
ChatGPT	20	74.20 ± 2.40	4.85 ± 0.37	18.60 ± 0.82
Gemini	20	76.00 ± 1.92	4.95 ± 0.22	19.35 ± 0.59
Copilot	20	67.85 ± 2.03	4.35 ± 0.49	17.10 ± 0.72
Overall p-value*	-	<0.001	<0.001	<0.001

Gemini achieved the highest educational quality scores across all assessment instruments, with mean DISCERN, GQS, and PPFERS scores of 76.00 ± 1.92, 4.95 ± 0.22, and 19.35 ± 0.59, respectively. ChatGPT also demonstrated high educational quality, with corresponding mean scores of 74.20 ± 2.40, 4.85 ± 0.37, and 18.60 ± 0.82. Copilot showed comparatively lower performance, with mean scores of 67.85 ± 2.03, 4.35 ± 0.49, and 17.10 ± 0.72, whereas the YouTube video cohort achieved mean scores of 66.24 ± 8.64, 4.28 ± 0.74, and 16.88 ± 1.96.

Overall group comparisons demonstrated statistically significant differences for all three assessment instruments. DISCERN scores differed significantly among groups (Kruskal-Wallis H = 40.53, p < 0.001), as did GQS scores (H = 23.27, p < 0.001) and PPFERS scores (H = 41.47, p < 0.001).

**Figure 2:**
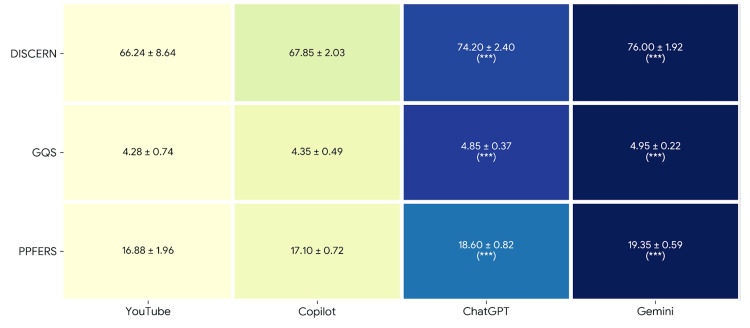
Summary matrix of the educational quality scores across different platforms The values within each cell represent the mean ± SD for DISCERN, GQS, and PPFERS. Color intensity reflects the performance hierarchy from lowest (light yellow) to highest (dark blue). * indicates a statistically significant superior performance (p < 0.001) compared to both the YouTube video cohort and Copilot, based on Dunn’s post-hoc test with Bonferroni correction. No statistically significant differences were observed between Gemini and ChatGPT, or between Copilot and the YouTube cohort (p > 0.05). SD: standard deviation, GQS: Global Quality Scale, PPFERS: Pediatric Pelvic Floor Education and Reliability Score

Pairwise post-hoc comparisons using Dunn’s test with Bonferroni correction revealed that Gemini and ChatGPT both achieved significantly higher DISCERN scores than the YouTube video cohort (p < 0.001 for both) and Copilot (p < 0.001 for both). Similarly, for GQS and PPFERS assessments, both Gemini and ChatGPT demonstrated statistically superior performance compared to the YouTube cohort (p < 0.001) and Copilot (p < 0.01). In contrast, no statistically significant differences were observed between Gemini and ChatGPT across all three evaluation instruments (p > 0.05). Furthermore, the differences between Copilot and the YouTube video cohort did not reach statistical significance for any of the metrics (p > 0.05) (Figure 2).

## Discussion

The present study evaluated and compared the educational quality, reliability, and safety-related content of YouTube videos and AI-generated responses related to pediatric pelvic floor dysfunction and its conservative management. The principal finding was that both information sources generally provided moderate-to-high quality educational content within the framework of the assessment tools used in this study. AI-generated responses, particularly those produced by Gemini and ChatGPT, achieved higher mean DISCERN, GQS, and PPFERS scores than the YouTube video cohort. However, these findings should not be interpreted as evidence of overall clinical superiority, real-world reliability, or safety of AI-generated information compared with YouTube content. Rather, they indicate that under standardized, question-based assessment conditions, some AI chatbots provided more structured, comprehensive, and pediatric urotherapy-oriented educational responses, as measured by the scoring instruments used in this study. From a real-world perspective, these findings are clinically relevant because families may use both YouTube videos and AI chatbots to guide home-based exercise practices, determine whether professional evaluation is necessary, and interpret clinician-provided urotherapy recommendations.

The superior performance of AI chatbots may be attributed to their ability to synthesize information from large volumes of medical literature and to generate structured responses that adhere to coherent educational frameworks [9,13]. In contrast to user-generated online content, AI systems tend to provide comprehensive explanations that simultaneously address symptoms, treatment principles, safety considerations, and indications for professional evaluation. In our study, Gemini achieved the highest scores across all evaluation instruments, followed closely by ChatGPT. These findings are consistent with recent studies evaluating AI-generated information in urology and urogynecology [10-12]. Deger et al. reported that large language model-based systems generated highly accurate and clinically useful responses for common urogynecological conditions, although occasional inaccuracies and omissions remained present [11]. Similarly, Kowalski and Brechtel emphasized the growing role of AI chatbots in urogynecology while highlighting the continued importance of expert supervision, clinical validation, and careful interpretation of AI-generated information [10]. Collectively, these studies suggest that modern AI systems may provide structured, patient-oriented educational information that may be useful; however, their outputs still require clinical oversight, contextual interpretation, and validation before use in pediatric urotherapy education [9-13].

Although the overall quality of YouTube videos was favorable, substantial variability was observed among individual videos. A notable finding was that 44% of included videos were uploaded by physiotherapists or pediatric physical therapists, which likely contributed to the relatively high mean DISCERN and GQS scores observed in the video cohort. Healthcare institutions also accounted for a substantial proportion of the uploaded content. Nevertheless, the educational quality of videos remained heterogeneous, as reflected by the presence of low-quality videos according to the PPFERS classification. This finding is clinically important because inappropriate pelvic floor exercise instruction may have unintended consequences in children with dysfunctional voiding. Current pediatric urotherapy principles emphasize coordinated pelvic floor relaxation, correct toilet posture, breathing techniques, and avoidance of maladaptive voiding behaviors [1,2]. Previous studies have demonstrated that structured urotherapy and pelvic floor rehabilitation can significantly improve symptoms of dysfunctional voiding and daytime urinary incontinence, highlighting the importance of correct exercise instruction and adherence to evidence-based treatment principles [16,17]. Educational resources that encourage breath-holding, excessive pelvic floor contraction, or repeated interruption of urinary flow may theoretically reinforce dysfunctional voiding patterns and reduce treatment effectiveness.

Our findings are broadly consistent with previous studies evaluating pelvic floor-related content on YouTube. Culha et al. reported substantial variability in the reliability and quality of pelvic floor muscle exercise videos. At the same time, Shah et al. demonstrated that video popularity was not necessarily associated with educational reliability [6,7]. More recently, Gunay et al. found that Kegel exercise videos on YouTube varied considerably in terms of accuracy, completeness, and patient guidance, highlighting the ongoing need for expert-reviewed educational resources [5]. Similarly, Cakir and Caglar reported heterogeneous quality across YouTube videos on pediatric urological diseases and emphasized the importance of careful content selection by patients, caregivers, and healthcare professionals [8]. Therefore, despite the generally acceptable quality of available YouTube content, the variability in educational quality underscores the need for careful content selection and professional guidance when families use online resources to manage pediatric pelvic floor dysfunction.

The findings of this study have several implications for clinical practice. AI chatbots demonstrated particular strengths in delivering structured, text-based educational guidance and consistently achieved high scores for reliability and guideline consistency. However, unlike video-based platforms, AI systems cannot currently provide visual demonstrations of exercise performance, body positioning, breathing techniques, or biofeedback-related activities. Consequently, the two educational modalities may be viewed as complementary rather than competing resources. Healthcare professionals may consider directing families to reputable AI-assisted educational tools while also recommending clinician-reviewed videos for visual instruction. The development of institutionally curated playlists or clinician-approved educational libraries may further improve the quality and safety of online patient education in pediatric pelvic floor dysfunction. In real-world clinical practice, these findings may help clinicians counsel families more specifically about how to use digital resources safely: AI chatbots may be useful for general explanations, question generation, and reinforcing written instructions, whereas video-based resources may be more appropriate for demonstrating posture, relaxation, and breathing techniques when they are clinician-reviewed. However, neither modality should be used as a stand-alone source for diagnosis, treatment planning, or exercise correction in children with pelvic floor dysfunction.

A major strength of the present study is its focus on pediatric pelvic floor dysfunction. This area has received considerably less attention than adult pelvic floor disorders in studies evaluating online health information. Furthermore, we are unaware of any previous studies that directly compare YouTube videos and AI chatbot responses regarding pediatric pelvic floor dysfunction using established assessment tools and a pediatric-specific educational scoring checklist. Additionally, the use of standardized evaluation instruments, together with a pediatric-specific assessment framework and formal nonparametric statistical comparisons, including appropriate post hoc correction for multiple testing, strengthens the study's methodological rigor. Finally, by evaluating both video-based and AI-generated educational resources, this study provides a comparative overview of currently accessible online information sources for parents and caregivers seeking guidance on pediatric pelvic floor dysfunction.

Several limitations should be acknowledged. First, only English-language content was evaluated, which may limit generalizability to other languages and healthcare settings. Second, pediatric pelvic floor dysfunction is a relatively specialized topic; therefore, the number of eligible pediatric-specific YouTube videos was limited. Third, the YouTube analysis was based on the first 50 videos retrieved for each search term using YouTube’s default relevance-based algorithm. Although this strategy was intended to reflect the easily accessible content users are likely to encounter during routine online searches, it may not fully capture all user behaviors, search preferences, or personalized recommendations. Additionally, YouTube search results are dynamic and may change over time due to algorithmic updates and shifts in content availability.

Fourth, comparisons between YouTube videos and AI-generated responses should be interpreted with caution because they represent different educational modalities and were evaluated using different observational units: unprompted long-form videos versus standardized text-based responses to curated questions. This structural asymmetry may have favored AI systems in scoring domains related to organization, completeness, and direct responsiveness to the assessment items. Therefore, the higher scores achieved by AI chatbots should be interpreted within the structured assessment framework of this study rather than as evidence of real-world educational superiority. Fifth, although AI systems achieved relatively high educational quality scores, the potential for hallucinations, incomplete responses, or inaccurate medical information remains an important concern. Moreover, exact backend model identifiers were not publicly displayed or consistently recorded across all chatbot platforms, particularly in free public interfaces, and chatbot performance may change over time due to model updates, platform modifications, and changes in default web-connected functions.

Sixth, although the chatbot questions were primarily developed according to pediatric urotherapy principles, relevant clinical guidance, and expert clinical judgment, common educational themes observed in YouTube videos were also considered to ensure topic relevance for caregivers and patients. This partial overlap may have introduced potential incorporation bias. Additionally, this study did not perform a direct item-by-item guideline-adherence analysis against ICCS recommendations. Therefore, statements regarding guideline consistency should be interpreted as structured content-based assessments rather than formal ICCS concordance analyses. Future studies should perform direct guideline-mapping analyses against ICCS recommendations to more precisely evaluate clinical accuracy and guideline concordance.

Seventh, although all materials were independently assessed by two reviewers and inter-rater reliability was quantified for all three assessment instruments, the scoring process still involved subjective judgment. Inter-rater reliability was excellent for DISCERN, moderate for GQS, and good for PPFERS. The moderate agreement observed for GQS indicates that some variability in global quality ratings may remain. Accordingly, GQS-related comparisons should be interpreted more cautiously than DISCERN and PPFERS findings, as GQS provides a broad, global judgment and may be more susceptible to subjective interpretation by reviewers.

Finally, the PPFERS was developed by the authors as a study-specific, pediatric-focused structured scoring checklist because no existing pediatric-specific tool adequately evaluates key components of conservative pelvic floor education, such as voiding posture, breathing synchronization, exercise instruction, warning signs requiring medical evaluation, and consistency with pediatric urotherapy principles. Although the checklist was developed based on ICCS recommendations, contemporary pediatric urotherapy guidance, expert clinical input, and pilot assessment, it has not undergone full formal psychometric validation. Inter-rater reliability for the total PPFERS score was assessed and found to be good (ICC = 0.84); however, external validation, construct validity testing, and responsiveness assessment were not performed. Because PPFERS items represent different formative content domains rather than interchangeable reflective items, internal consistency was not considered the primary reliability metric; therefore, findings based on PPFERS should be interpreted as exploratory pediatric-specific content assessments rather than as results derived from a fully validated scale.

## Conclusions

Both YouTube videos and AI-generated responses provided generally useful educational information regarding pediatric pelvic floor dysfunction within the assessment framework used in this study. AI chatbot responses, particularly those generated by Gemini and ChatGPT, achieved higher reliability and educational quality scores than YouTube videos and appeared more structured and consistent with contemporary pediatric urotherapy principles. However, these findings should not be interpreted as evidence of overall clinical superiority, real-world clinical effectiveness, or complete safety of AI-generated information. Rather, they suggest that AI-assisted educational tools may serve as valuable adjuncts to patient and caregiver education when used under appropriate professional guidance. AI systems and online videos should therefore be regarded as supportive educational resources rather than as substitutes for professional medical assessment, individualized treatment planning, clinician-supervised urotherapy, or hands-on instruction that requires visual demonstration, posture correction, breathing coordination, and exercise performance monitoring.

## Appendices

**Table 4:** Standardized questions used for AI chatbot evaluation AI: artificial intelligence

Question no.	Standardized question
Q1	What are pelvic floor exercises for children?
Q2	Can pelvic floor exercises help children with dysfunctional voiding?
Q3	Can pelvic floor exercises help bedwetting in children?
Q4	How can a child correctly identify the pelvic floor muscles?
Q5	How often should children perform pelvic floor exercises?
Q6	What mistakes should children avoid during pelvic floor exercises?
Q7	Should children stop urinating midstream to practice pelvic floor exercises?
Q8	Is holding the breath during pelvic floor exercises recommended?
Q9	Can pelvic floor exercises replace medical treatment for urinary problems in children?
Q10	When should a child with urinary symptoms be evaluated by a healthcare professional?
Q11	Can pelvic floor exercises help daytime urinary incontinence?
Q12	Can pelvic floor exercises help constipation-related urinary symptoms?
Q13	Are pelvic floor exercises safe for all children?
Q14	Can pelvic floor exercises be performed without physiotherapist supervision?
Q15	What is the best advice for parents searching online for pelvic floor exercise information for children?
Q16	What is dysfunctional voiding in children?
Q17	What is bladder training and how is it used in children?
Q18	What are the warning signs that require medical evaluation in children with urinary symptoms?
Q19	Can pelvic floor exercises worsen symptoms if performed incorrectly?
Q20	What is the difference between pelvic floor exercises and urotherapy?

**Table 5:** PPFERS scoring criteria Total score: 0-20 points. Higher scores indicate greater educational quality, reliability, safety, and consistency with contemporary pediatric urotherapy principles. PPFERS: Pediatric Pelvic Floor Education and Reliability Score

Domain	Score = 0	Score = 1	Score = 2
Scientific accuracy	Contains incorrect or misleading medical information	Partially accurate but incomplete information	Scientifically accurate and evidence-based information
Child-friendly explanation	No child-oriented explanation	Partially adapted for children or caregivers	Clear, age-appropriate, and easily understandable explanation
Correct exercise instruction	Incorrect or potentially misleading exercise instructions	Basic exercise description with limited guidance	Correct and comprehensive exercise instructions consistent with pediatric practice
Avoidance of potentially harmful recommendations	Includes potentially harmful advice (e.g., repeated interruption of urinary stream or inappropriate contraction techniques)	Potentially harmful issues not clearly addressed	Explicitly avoids harmful recommendations and provides safe guidance
Breathing guidance	No mention of breathing	Brief or incomplete mention	Appropriate breathing techniques clearly explained
Exercise frequency recommendations	No recommendation	General recommendation without practical details	Clear, practical, and appropriate frequency recommendations
Relevance to pediatric urinary symptoms	Not specific to pediatric urinary symptoms	Limited discussion of pediatric symptoms	Clearly addresses pediatric urinary symptoms and their management
Recommendation for medical evaluation when appropriate	No recommendation to seek medical care	Vague recommendation	Clearly advises medical evaluation when warning signs or persistent symptoms are present
Consistency with guideline-based pediatric urotherapy principles	Inconsistent with accepted pediatric urotherapy principles	Partially consistent	Fully consistent with current guideline-based pediatric urotherapy principles
Overall educational usefulness	Minimal educational value	Moderately useful educational content	Highly useful, comprehensive, and clinically appropriate educational content
